# Multidimensional evaluation of nutritional security policies in the Republic of Moldova: gaps, progress, and alignment with international standards

**DOI:** 10.3389/fpubh.2025.1522097

**Published:** 2025-01-30

**Authors:** Rodica Siminiuc, Dinu Țurcanu, Sergiu Siminiuc

**Affiliations:** ^1^Faculty of Food Technology, Technical University of Moldova, Chisinau, Moldova; ^2^Faculty of Computers, Informatics and Microelectronics, Technical University of Moldova, Chisinau, Moldova

**Keywords:** nutritional security, public policies, educational policies, labeling policies, fiscal policies, Republic of Moldova, healthy diets

## Abstract

**Introduction:**

Nutritional security is increasingly recognized as a critical component of public health, particularly in addressing the growing burden of non-communicable diseases such as obesity, diabetes, and cardiovascular disease. In the Republic of Moldova, rapid changes in food availability and consumption patterns have intensified the need for robust public policies to promote healthy dietary habits.

**Methods:**

This study evaluates Moldova’s public policies on nutritional security using a multidimensional model adapted from the Policy Evaluation Network framework. The model assesses five key policy categories: educational, strategic, labeling, monitoring, and fiscal policies, through a comprehensive 28-indicator structure.

**Results:**

The findings indicate that Moldova’s policy efforts in nutritional security are at a “satisfactory” level, with foundational strategies in place but significant gaps in policy cohesion, implementation, and monitoring. Educational and strategic policies show moderate progress, yet labeling, monitoring, and fiscal policies are underdeveloped, limiting their impact on public health.

**Discussion:**

The results emphasize the need for a more coordinated, multi-sectoral approach, incorporating targeted fiscal incentives, systematic monitoring frameworks, and comprehensive educational reforms.

## 1 Introduction

Nutritional security has emerged as a global public health priority, given the significant role of poor dietary habits in the rise of non-communicable diseases (NCDs) such as cardiovascular disease, diabetes, and obesity (1, 2). In low-and middle-income countries, where shifts in food availability and consumption patterns are rapid, the public health impact of diet-related issues is even more pronounced (3). The Republic of Moldova exemplifies this trend; as dietary shifts evolve and access to a diverse range of food products increases, addressing nutritional security through robust public policies becomes essential to protect population health and encourage healthier lifestyles.

Moldova has taken preliminary steps to improve public health through various nutrition-focused policies, including initiatives in education, dietary behavior, and NCDs prevention. However, these efforts remain fragmented, under-resourced, and lack the comprehensive structure observed in European Union (EU) countries (2, 4–7). While Moldova’s framework includes elements like education, labeling, and strategic plans, it does not yet encompass the multifaceted, coordinated approach needed to fully support nutritional security.

Such a policy structure should integrate regulatory, fiscal, and monitoring measures to create an environment where food choices are both aligned with public health goals and accessible to the entire population.

Research underscores the value of a multidimensional approach to nutritional security—one that combines education, affordability, and accessibility to support long-term healthy eating behaviors sustainably. Countries employing such models often implement policies like front-of-pack labeling, taxes on sugar-sweetened beverages, and subsidies for nutritious foods, which have proven effective in reducing diet-related diseases (3, 8–10). For Moldova, a similar approach equires cross-sectoral collaboration involving government, private industry, and civil society to ensure a unified and effective response to nutritional challenges.

This study evaluates Moldova’s current public policies on nutritional security using a multidimensional model that examines five key areas: educational, strategic, labeling, monitoring, and fiscal policies. By analyzing the strengths and weaknesses of each policy category, this assessment provides a comprehensive view of Moldova’s progress and the specific areas needing improvement. A key novelty of this research lies in its application of an integrated evaluation framework uniquely adapted to Moldova’s context. Unlike previous studies, which may focus on isolated aspects of nutritional policy, this approach consolidates various policy components, providing an actionable, evidence-based roadmap for policy enhancement.

The importance of this study is threefold. First, it identifies specific policy areas that directly impact public health outcomes, offering insights that can shape governmental strategies to reduce the burden of NCDs and other diet-related conditions. Second, by aligning Moldova’s policy evaluation with global best practices, this research bridges the gap between current efforts and standards recommended by leading organizations such as the World Health Organization (WHO) and the European Food Safety Authority (EFSA). Finally, this study contributes to the limited body of literature on nutritional security in Moldova, establishing a foundation for future research, policy development, and monitoring.

In summary, this research highlights the pivotal role of public policies in shaping a secure nutritional environment and offers a pathway for Moldova to strengthen its public health strategies. By addressing the identified gaps and adopting a comprehensive, data-informed approach, Moldova can move toward a healthier and more resilient population, supporting its broader social and economic development goals. The findings and recommendations from this study aim to guide policymakers, public health officials, and stakeholders in building a robust, sustainable framework for nutritional security in Moldova.

## 2 Research methodology

To assess the level of nutritional security in the Republic of Moldova, this study used the *Policy Evaluation Network* (PEN) model, an evaluation framework for public policies applied in the “Healthy Diet for a Healthy Life” program. This program monitors health-related behaviors in Europe and includes recognized indicators for evaluating nutrition and physical activity policies. The PEN model was selected due to its broad applicability in analyzing nutrition policies, especially in terms of content, implementation, and effectiveness (11, 12).

### 2.1 Indicator selection and categories of nutrition policies

To construct the multifactorial evaluation model, 28 evaluation indicators were selected and organized into five main categories of nutrition policies:

- Educational policies – relating to nutrition education in schools and medical institutions, dietary guidelines, and their periodic evaluation;
- Strategic policies – addressing national nutritional health through strategies and action plans for preventing and controlling NCDs;
- Labeling policies – including regulations for ingredient labeling, nutrition declarations, and front-of-pack labeling systems;
- Monitoring policies – aimed at monitoring the nutritional status of the population, food consumption, and health promotion through food marketing;
- Fiscal policies – governing taxes and subsidies to promote a healthy food basket.

The selected indicators were classified based on their relevance to each policy category, and each indicator was assigned a weight according to the PEN evaluation model.

### 2.2 Scoring and interpretation methodology

The applied evaluation model uses a binary scoring system for each item, awarding one point for each affirmative answer (“Yes = 1”) and zero points for negative answers (“No = 0”). The total score for each policy category was calculated by adding the obtained scores and comparing them with the maximum possible score for that category. The total score was calculated as follows:


$$\mathrm{Score=}\Sigma P_{I}/\Sigma T_{I}\times 100\text{,}$$

Where:

PI – represents the number of items with positive responses,

TI – represents the total number of items.

The scores were interpreted using the following intervals to determine the level of nutritional security:

- 0–20%: Very low level of nutritional security,
- 21–40%: Satisfactory level of nutritional security,
- 41–60%: Good level of nutritional security,
- 61–80%: Very good level of nutritional security,
- 81–100%: Excellent level of nutritional security.

This scoring methodology enabled a clear and systematic evaluation of existing policies and identified gaps and shortcomings in the implementation and monitoring of nutritional security policies in the Republic of Moldova.

The adopted methodology provides a comprehensive and replicable framework, suitable for analyzing the diversity of nutrition policies and their impact on food and nutritional security. The model is flexible enough to be applied in evaluating similar policies in other regions and allows easy adaptation of criteria based on national specifics. This methodological framework also contributes to understanding how each policy category can be improved to enhance the effectiveness of measures taken to ensure optimal nutritional security.

## 3 Results

### 3.1 Educational policies

The assessment of educational policies revealed several gaps in implementing comprehensive nutrition education in Moldova’s educational and medical institutions. Five key indicators were analyzed to evaluate the implementation of these policies, and the results indicate a limited integration of nutrition-related content in national educational curricula and professional training programs (3, 13, 14) (Table 1).

**Table 1:** Key indicators in educational policies on nutritional security.

No	Items	Implemented (1)	Not Implemented (0)
1.	Does a Nutrition Education Curriculum exist for all health professionals, consisting of at least one module equivalent to 5 European Credit Transfer and Accumulation System (ECTS) credits?		No
2.	Does the school curriculum include nutrition education as a standard component?	Yes	
3.	Is nutrition education mandatory in the school curriculum across different education levels?		No
4.	Has the government officially published and implemented national dietary guidelines?	Yes	
5.	Does the government have plans to re-evaluate the dietary guidelines on a timely basis (usually every 5 years)?		No
The score		2	

Yes = 1 point (Implemented), No = 0 points (Not Implemented). The total score reflects the sum of implemented responses for all items.

Nutrition science is largely missing from the core curriculum of medical programs in Moldova. Only one specialty includes a mandatory nutrition course with 4 ECTS credits, while nutrition remains an elective subject in other fields. At the primary and secondary school levels, the curriculum includes optional health education modules covering basic nutrition topics. However, this education remains optional rather than compulsory, and the allocated hours (6 for grades V-VI, 5 for grades VII-IX, and 4 for grades X-XII) are insufficient to provide a comprehensive understanding of nutrition.

Although some nutrition-related content exists within school programs, nutrition education is not consistently mandatory across all educational levels. The Moldovan government has developed dietary guidelines under the initiative of the United Nations, with the publication of the “Rational Nutrition, Food Safety, and Changing Eating Behavior” guide in 2019. This guide includes general recommendations but lacks detailed guidance on specific population needs. While these guidelines exist, there is currently no systematic process to review and update these guidelines periodically, as recommended internationally. Regular evaluation and updates, ideally every 5 years, are essential to incorporate the latest scientific findings and adapt recommendations to evolving public health needs.

### 3.2 Strategic policies

The evaluation of strategic policies for nutritional security in the Republic of Moldova focused on ten key indicators aimed at assessing the presence and implementation of national strategies, regulations, and support programs related to nutrition and NCDs prevention (Table 2). The results reveal both progress and notable gaps in the alignment of these policies with international standards and best practices.

**Table 2:** Key indicators in strategic policies on nutritional security.

No	Items	Implemented (1)	Not Implemented (0)
1	Does the government have a national strategy or action plan aimed at improving nutrition and reducing the burden of non-communicable diseases (NCDs) in both children and adults?	Yes	
2	Are there government-run food assistance programs to support vulnerable populations?	Yes	
3	Do social support programs ensure access to healthy food and establish clear nutrition standards?		No
4	Are there regulations governing the sale of food and non-alcoholic beverages to children within schools or near school premises?		No
5	Are there government regulations supporting meal provision, school food programs, fruit and vegetable initiatives, or supplementary milk programs for school children?	Yes	
6	Has the government established specific targets for nutrient intake to align with the dietary recommendations of the World Health Organization (WHO) and the European Food Safety Authority (EFSA) at the national level?	Yes	
7	Does the government implement a system or strategy with mandatory standards for the nutrient content of industrially processed foods?	Yes	
8	Has the government implemented specific measures to ban or effectively eliminate industrial trans fats?	Yes	
9	Does the country have a national food composition table or a comprehensive food composition database?		No
10	Are there government mechanisms in place to oversee, coordinate, and harmonize nutrition actions, such as a National Nutrition Council, Technical Working Group, or Steering Committee?		No
The score		6	

Yes = 1 point (Implemented), No = 0 points (Not Implemented). The total score reflects the sum of implemented responses for all items.

The Republic of Moldova has implemented a national program for the prevention and control of priority NCDs (2023–2027), targeting conditions such as cardiovascular diseases, cancer, diabetes, and chronic respiratory diseases. This program provides a foundation for addressing nutrition-related health issues. The government has established social support food programs, such as Social Assistance Canteens (SAC), to provide free meals to vulnerable groups. However, these programs lack standardized nutrition criteria and a national framework to regulate the nutritional quality of food provided. SAC providers often rely on generic caloric norms from educational institutions or available food supplies, leading to inconsistent dietary quality across different providers (6, 42). Currently, the sale of food and beverages near educational institutions is minimally regulated. While past legislation aimed to limit access to unhealthy foods within a 100-meter radius around schools, this restriction was repealed in 2016 (5).

The Ministry of Health has established recommendations for school meals, encouraging whole foods, fruits, and vegetables while limiting foods high in sugar, fat, and salt. The government has not established clear national targets for nutrient intake in alignment with WHO and EFSA recommendations. While some macronutrient ratios are outlined in national dietary guidelines, detailed nutrient-specific targets are lacking. The Republic of Moldova has adopted EU regulations that require labeling of salt content in processed foods, but mandatory standards for nutrient content in other processed foods remain limited.

In alignment with international recommendations, the government has proposed regulations limiting the allowable content of trans fats in processed foods to a maximum of 2 grams per 100 grams of fat. Efforts to establish a national food composition database have been initiated, with data on approximately 1,600 food products collected from local markets. However, the database requires further expansion, validation, and regular updates to serve as a reliable resource for nutritional policy development and dietary recommendations. Currently, there is no centralized mechanism or council for coordinating nutrition-related actions across various sectors.

Out of the ten indicators assessed in the strategic policy category, six received positive responses, resulting in a score of 6 out of a possible 10 points. This score suggests moderate progress in implementing strategic policies for nutritional security, but also highlights significant areas for improvement. Strengthening regulatory frameworks, establishing clear nutritional targets, and enhancing intersectoral coordination would considerably improve the effectiveness of strategic policies in Moldova, supporting healthier dietary patterns and reducing the burden of nutrition-related diseases in the population. By addressing these areas, Moldova can advance toward a more comprehensive and cohesive national strategy for nutritional security that aligns with international best practices.

### 3.3 Labeling policies

The assessment of labeling policies in the Republic of Moldova focused on five key indicators to determine the effectiveness of food labeling regulations in guiding consumers toward healthier dietary choices. The results (Table 3) indicate that while some progress has been made, there are notable gaps in implementing comprehensive labeling standards that align with international best practices.

**Table 3:** Key indicators in labeling policies on nutritional security.

No	Items	Implemented (1)	Not Implemented (0)
1	Have regulations been adopted to mandate ingredient lists and nutrient declarations on the labels of all packaged foods, in alignment with Codex recommendations?	Yes	
2	Are there evidence-based regulations established to approve and periodically review food claims?		No
3	Are the criteria for nutrition or health claims defined in any legislation, regulation, or official guidance?	Yes	
4	Has front-of-pack labeling been introduced through legislation, regulations, or official guidelines?		No
5	Does the government provide guidelines for a simple and clear labeling system on chain restaurant menu boards to help consumers understand the nutrient quality and energy content of the food offered?		No
The score		2	

Yes = 1 point (Implemented), No = 0 points (Not Implemented). The total score reflects the sum of implemented responses for all items.

Moldova has implemented regulations that require all packaged food products to display a list of ingredients and a nutritional declaration, following Codex Alimentarius recommendations. This policy helps consumers make informed choices by providing clearer transparency regarding the content of packaged foods. At present, there are no national regulations specifically designed to evaluate and review food claims, such as health or nutritional claims, on product labels. The criteria for nutrition and health claims on food products are partially regulated under Government Decision no. 196, which sets basic standards for such claims (15–17).

Moldova currently lacks a FOPL system, such as color-coded or symbol-based labels, which have been shown to help consumers quickly assess the nutritional quality of foods. There is no government-mandated system for displaying nutritional information on menus in chain restaurants or food service establishments.

Out of the five indicators assessed in the labeling policy category, only two received positive responses, resulting in a score of 2 out of a possible 5 points. This score reflects the need for more robust and comprehensive labeling policies to promote nutritional transparency and guide consumer choices effectively. Implementing a front-of-pack labeling system, establishing stronger criteria for health and nutrition claims, and expanding labeling requirements to chain restaurants would significantly enhance the impact of Moldova’s labeling policies on public health. By addressing these areas, Moldova could strengthen its regulatory framework for food labeling, helping consumers make healthier choices and reducing the risk of diet-related diseases in the population.

### 3.4 Monitoring policies

The assessment of monitoring policies for nutritional security in the Republic of Moldova focused on seven key indicators to evaluate the extent of systematic tracking, evaluation, and oversight of food environments and nutritional health. The results indicate that Moldova’s monitoring framework is currently underdeveloped, with significant gaps in data collection, analysis, and policy effectiveness assessment (Table 4).

**Table 4:** Key indicators for monitoring nutritional security policies.

No	Items	Implemented (1)	Not Implemented (0)
1	Are there government systems in place for systematically monitoring food environments at local, regional, and national levels?		No
2	Has any work been conducted or planned to track progress in improving the nutrient content of various products over time?	Yes	
3	Has any research been conducted to assess the extent and characteristics of food marketing at the national level through studies or surveys?		No
4	Have any activities been conducted or planned to carry out food consumption and nutrient intake surveys?	Yes	
5	Are there policies, strategies, or systematic plans in place to monitor the nutritional status of both adults and children?		No
6	Has any work been conducted or planned to assess the overall effectiveness of nutrition programs or policies, such as through impact studies, process evaluations, or cost-effectiveness analyses?		No
7	Is progress in reducing health inequalities, addressing the health impacts on vulnerable populations, and monitoring the social and economic determinants of health regularly evaluated?		No
The score		2	

Yes = 1 point (Implemented), No = 0 points (Not Implemented). The total score reflects the sum of implemented responses for all items.

Currently, the Republic of Moldova lacks a centralized, government-led system for the comprehensive monitoring of food environments at local, regional, and national levels. However, the National Food Safety Agency has implemented annual monitoring plans to assess certain specific nutrient contents, such as iron and folic acid in flour and bakery products. At the national level, there is no organized effort to assess the extent and nature of food marketing, particularly regarding unhealthy food products targeted at children. Although surveys like the FEEDcities project have shed light on dietary patterns in specific contexts, such as street food in Chisinau, the absence of large-scale, representative data on food consumption and nutrient intake limits the ability to address broader public health needs.

Moldova does not have an organized system for systematically monitoring the nutritional status of adults and children. Current data on diet-related health conditions and nutritional deficiencies are outdated, with the last major study conducted with United Nations International Children’s Emergency Fund (UNICEF) support in the 1990s. Moreover, no formal evaluation has been conducted to assess the impact, cost-effectiveness, or process quality of nutrition programs and policies in Moldova. Currently, there is no national framework for monitoring of health inequalities or the nutritional status of vulnerable populations, nor is there a system to track the social and economic determinants of health related to nutrition.

Out of the seven indicators assessed in the monitoring policy category, only two received positive responses, resulting in a score of 2 out of a possible 7 points. This score underscores the critical need for comprehensive and structured monitoring policies in Moldova. By establishing a robust, national system to systematically track food environments, nutrient intake, and health outcomes, Moldova could improve the evidence base for nutrition policies and better address the nutritional needs of its population.

### 3.5 Fiscal policies

The evaluation of fiscal policies in the Republic of Moldova aimed at promoting healthier food choices focused on four key indicators to assess the effectiveness of fiscal measures in encouraging nutritious diets and discouraging the consumption of less healthy foods (Table 5).

**Table 5:** Key indicators in fiscal policies on nutritional security.

No	Items	Implemented (1)	Not Implemented (0)
1	Have fiscal policies been implemented to discourage the consumption of unhealthy food and beverages and promote healthier alternatives?		
1.1.	Have tax policies been implemented to introduce or increase levies on unhealthy food and beverages?		No
1.2.	Have tax policies been implemented to eliminate or reduce taxes on healthier food and beverages?		No
1.3	Have fiscal policies been implemented to introduce or increase subsidies for healthier food and beverages?		No
1.3.	Have fiscal policies been established to eliminate subsidies for unhealthy food and beverages?		No
The score		0	

Yes = 1 point (Implemented), No = 0 points (Not Implemented). The total score reflects the sum of implemented responses for all items.

The findings indicate a significant lack of comprehensive fiscal policies targeting nutrition, which limits the government’s ability to influence dietary behaviors through economic incentives (18).

At present, the Republic of Moldova has not implemented any taxes specifically targeting unhealthy foods or sugar-sweetened beverages. Additionally, Moldova lacks policies to reduce taxes on healthier food options, such as fruits, vegetables, or whole grains. Furthermore, there are currently no subsidies in place to support the production or purchase of healthier foods. Moldova does not have a policy framework for removing existing subsidies on unhealthy food products.

Out of the four indicators assessed in the fiscal policy category, none received positive responses, resulting in a score of 0 out of a possible 4 points. This result highlights the urgent need for Moldova to develop fiscal policies that can actively promote healthy dietary choices and reduce the consumption of less nutritious foods. Introducing taxes on unhealthy foods, reducing taxes on nutrient-dense options, and implementing targeted subsidies would create a fiscal environment that supports nutritional security and encourages healthier eating habits.

### 3.6 Evaluation of public policy scores using the multidimensional model

**Figure 1:**
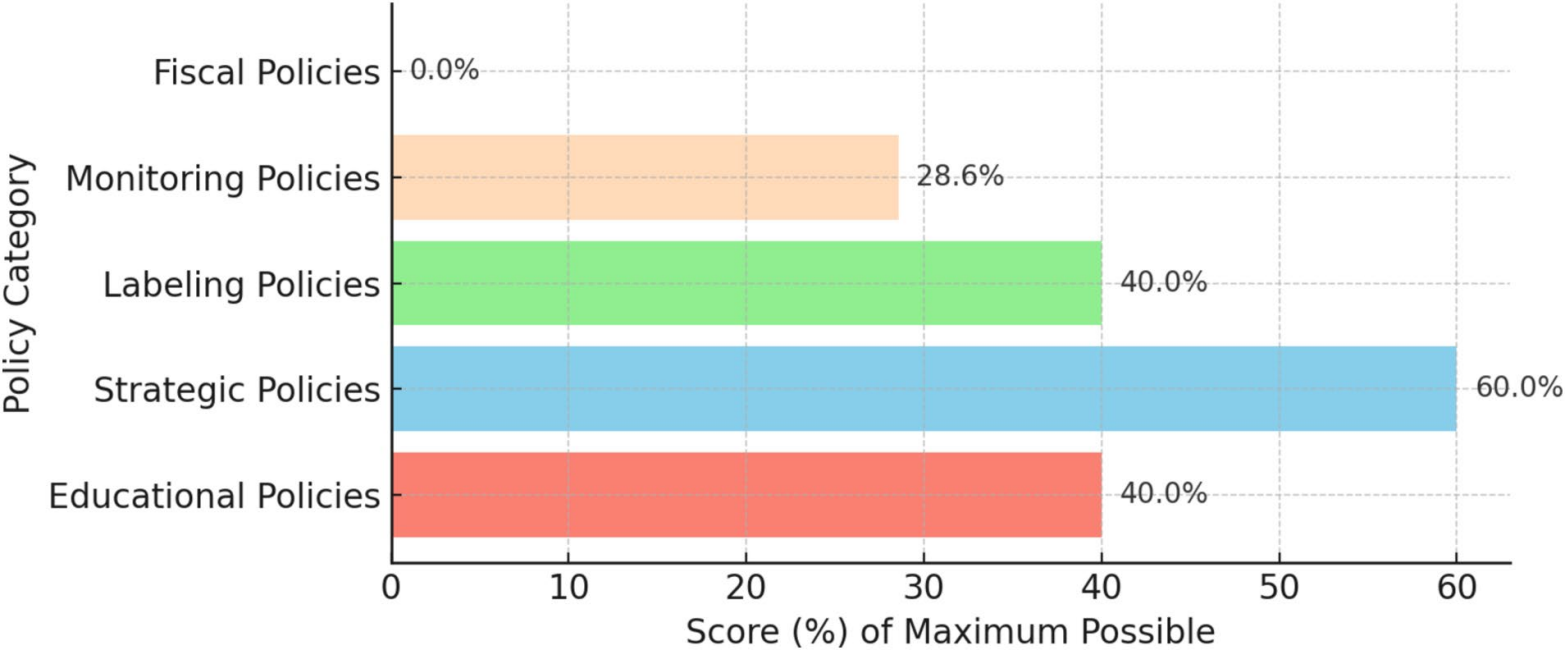
Performance of public policies on nutritional security in Moldova by category.

The developed multidimensional model evaluated the Republic of Moldova’s public policies on nutritional security across five key policy categories: educational, strategic, labeling, monitoring, and fiscal policies. Each category was scored based on its alignment with international standards and its effectiveness in promoting nutritional security. The results provide a comprehensive view of the strengths and weaknesses within Moldova’s policy framework, highlighting areas that require enhancement for better public health outcomes (Figure 1).

Educational policies: out of a possible 5 points, educational policies scored 2 points, reflecting limited integration of nutrition education at various levels and the need for a more structured and compulsory curriculum in schools and medical training programs.

Strategic policies: this category achieved a score of 6 out of 10 points, indicating moderate progress. Although some strategic policies are in place, such as a national NCDs prevention plan, there is a need for more robust regulations, clear nutrient intake targets, and improved coordination among sectors.

Labeling policies: with a score of 2 out of 5 points, labeling policies show some initial steps, such as mandatory ingredient lists and nutritional declarations, but lack comprehensive systems like front-of-pack labeling and menu labeling in restaurants, which could guide consumers toward healthier choices.

Monitoring policies: monitoring policies received 2 out of 7 points, indicating substantial gaps. Moldova lacks a cohesive system for tracking food environments, nutrient intake, and health outcomes across the population. Establishing a structured monitoring framework is essential for data-driven policy adjustments.

Fiscal policies: fiscal policies scored 0 out of 4 points, as Moldova currently has no fiscal measures to promote healthier food choices or discourage the consumption of unhealthy foods. Implementing taxes on sugar-sweetened beverages and subsidies for nutritious foods would align fiscal policies with public health objectives.

Across all categories, Moldova’s public policies on nutritional security achieved a cumulative score of 12 out of 31 points, which corresponds to 39%. This places Moldova’s overall policy performance in the “satisfactory” range, indicating a foundational but underdeveloped policy framework for nutritional security.

Recommendations for Policy Enhancement. The results suggest several priority areas for improvement:

- Expand nutrition education across schools and healthcare training programs to build a stronger foundation for public health.
- Strengthen strategic policies by setting clear, measurable nutrient intake targets and establishing mechanisms for policy coordination.
- Enhance labeling policies with the introduction of front-of-pack and menu labeling to simplify nutritional information for consumers.
- Develop monitoring systems to systematically track dietary patterns, nutritional health outcomes, and food marketing practices.
- Implement fiscal policies that use taxes and subsidies to create economic incentives for healthier food choices.

By addressing these gaps, Moldova can create a more comprehensive and effective policy framework for nutritional security that better aligns with international standards and supports the health of its population.

## 4 Discussion

The results of this study highlight both progress and significant gaps in the framework of public policies for nutritional security in the Republic of Moldova. While some advances have been made, particularly in educational and strategic policies, there are major deficiencies in areas such as fiscal measures, comprehensive monitoring, and food labeling.

### 4.1 Educational policies: strengthening nutrition education

The assessment of educational policies reveals significant gaps in integrating nutrition education into the school and medical curricula in Moldova. While the inclusion of a mandatory 4 ECTS credit course in one medical specialty is a step forward, it falls short of international standards, which recommend at least 5 credits for a comprehensive understanding of clinical nutrition (19).

In schools, existing optional modules and the limited hours allocated to nutrition topics are insufficient to influence long-term dietary behaviors. Studies show that early interventions significantly contribute to reducing risks associated with non-communicable diseases (20, 21). The introduction of a mandatory, well-structured nutrition education curriculum at all levels is essential.

Additionally, the dietary guidelines developed by the Moldovan government remain too general and lack detailed recommendations tailored to different population groups. The absence of a structured mechanism for periodic revisions reduces their relevance and effectiveness. Regular updates aligned with evolving scientific evidence are crucial for supporting evidence-based interventions (22).

### 4.2 Strategic policies: enhancing policy coordination and target setting

Strategic policies for preventing NCDs provide a solid but insufficiently coordinated foundation. Adopting a framework with clear objectives for nutrient intake aligned with international standards would enhance the effectiveness of existing strategies. For instance, the EU regulations limiting trans fats to 2 grams per 100 grams of fat represent an effective measure that could be implemented in Moldova (23).

The lack of a coordinating body, such as a National Nutrition Council, hinders the cross-sectoral collaboration required for policy implementation. Establishing such a body would facilitate cooperation among authorities, educational institutions, and industry, improving policy alignment and execution (24–27).

### 4.3 Labeling policies: improving transparency for healthier choices

Food labeling policies in Moldova are limited to ingredient lists and nutritional declarations, lacking a standardized FOPL system. Introducing simplified labeling systems, such as traffic light labels or Nutri-Score, could facilitate informed decision-making by consumers (28, 29).

Another critical aspect is nutrition and health claims. Without stringent regulations and validation mechanisms, consumers risk being misled. Strengthening these regulations would enhance trust and support healthier choices (30–32).

### 4.4 Monitoring policies: building a comprehensive data system

The lack of a comprehensive monitoring system limits Moldova’s ability to evaluate the impact of nutrition policies. For example, while annual plans exist to assess specific nutrients, they are insufficient to provide a complete picture of nutritional status (33).

Periodic assessments of nutritional status and dietary intake could guide targeted interventions and enable data-driven policy adjustments. Additionally, monitoring food marketing practices, especially for nutrient-poor products, is essential to reduce their negative influence on dietary behaviors (34, 35).

### 4.5 Fiscal policies: leveraging economic tools for public health

The absence of fiscal policies promoting healthy diets represents a missed opportunity for Moldova. Evidence shows that taxing sugar-sweetened beverages and calorie-dense, nutrient-poor foods, along with subsidizing nutrient-rich products, can reduce consumption and generate revenue for public health initiatives (36–38). Implementing such measures would contribute to creating a more favorable food environment and support the adoption of healthier eating habits, particularly among vulnerable populations.

### 4.6 Policy implications and future directions

The findings of this evaluation underscore the importance of a coordinated and multi-sectoral approach to improving nutritional security. Moldova’s progress in areas like education and strategic initiatives provides a foundation for developing more comprehensive policies. Key priorities for future development include:

- Institutionalizing a National Nutrition Council to facilitate coordination across government agencies, private sector stakeholders, and civil society.
- Implementing nationwide dietary surveys and health status monitoring to enable regular, evidence-based policy adjustments.
- Introducing fiscal policies aligned with public health objectives, which can serve as economic tools to encourage healthier dietary patterns.

By addressing these recommendations, Moldova has the opportunity to build a more resilient public health framework aligned with international standards, reducing the burden of diet-related diseases and promoting healthier lifestyles for its population. With continued policy refinement and commitment to data-informed decisions, Moldova can progress toward a more sustainable and health-promoting nutritional security environment, ultimately supporting the well-being of its citizens and future generations.

Policymakers have an impactful role in promoting healthy and sustainable food practices as they are involved in designing and implementing interventions such as farmer incentives, multilateral agreements, mandatory food labeling, reformulation or food taxes, etc. To carry out a current analysis of the progress and trends in the field of food security, particularly regarding nutritional security in the Republic of Moldova, several national policies, strategies and programs were studied. National documents and strategies were identified and analyzed, which it is assumed should also directly or indirectly address the nutrition dimension. It appears that nutrition, as an essential component of the food and nutrition security, lacks clear institutional oversight, with responsibilities for developing, implementing, and monitoring nutrition policies being fragmented and inadequately defined across various entities. The Food Security Strategy in the Republic of Moldova 2023–2030 does not address the “Food Utilization” dimension at all (25).

- The national development plan for the years 2023–2025 is the primary document that establishes the Government’s public policy priorities for 3 years and contains the priority directions and measures for the implementation of the country’s strategic objectives (25). In that plan, nutrition can only be found in creating a solid and inclusive social protection system to prevent malnutrition in kindergarten and nursery school children (39).
- The government activity program “Prosperous, safe, European Moldova,” approved by Parliament Decision No. 28 of February 16, 2023, has no nutritional goals (40).
- In the National Health Policy, the objectives outlined in 2007 are well-intentioned and remain relevant today. However, they require concretization regarding the monitoring of established goals, as well as reformulations, considering the progress and setbacks in the fields of health, nutrition, and well-being [(41), p. 886].

## 5 Conclusion

This study provides a comprehensive evaluation of public policies on nutritional security in the Republic of Moldova, employing an innovative multidimensional model adapted to the national context. The findings underscore both the progress made and the critical gaps that persist in developing coherent and effective policies to improve public health outcomes.

The analysis highlights the potential of a multidimensional approach in assessing the complexity of nutritional policies and identifying areas that demand urgent attention. By integrating diverse indicators across educational, labeling, monitoring, strategic, and fiscal domains, the study offers a holistic perspective on policy performance. It reveals foundational efforts in education and strategic initiatives while exposing significant shortcomings in fiscal measures, systematic monitoring, and transparent labeling systems.

The study makes a significant contribution to the literature by applying an evidence-based framework that aligns with international standards, providing actionable insights for policymakers. Recommendations such as implementing front-of-pack labeling systems, introducing fiscal incentives for healthier dietary choices, and establishing a centralized monitoring mechanism have practical implications for reducing diet-related disease burdens. These steps are critical for fostering a sustainable nutritional environment and improving population health.

Future research and policy efforts should focus on creating an integrated and coordinated approach to nutritional security, underpinned by robust intersectoral collaboration. Establishing a National Nutrition Council and adopting regular, evidence-informed policy reviews would ensure that Moldova can meet its public health objectives while aligning with global best practices. This work not only addresses the unique challenges of Moldova’s nutritional landscape but also sets a precedent for similar evaluations in other resource-constrained settings.

Through commitment to reform and evidence-based policymaking, Moldova can advance its public health framework, promoting healthier lifestyles and sustainable development for future generations.

## Data Availability

The original contributions presented in the study are included in the article/supplementary material, further inquiries can be directed to the corresponding author.
